# MR Imaging in Neurocritical Care

**DOI:** 10.5005/jp-journals-10071-23186

**Published:** 2019-06

**Authors:** Anurima Patra, Amit Janu, Arpita Sahu

**Affiliations:** 1-3 Department of Radiodiagnosis, Tata Memorial Hospital, Homi Bhabha National Institute, Mumbai, Maharashtra, India

## Abstract

**How to cite this article:** Patra A, Janu A, Sahu A. MR Imaging in Neurocritical Care. Indian J Crit Care Med 2019;23(Suppl 2):S104–S114.

As neurointensive care units are growing, neurocritical care, a subspecialty, requires an extensive knowledge of neuroanatomy, neurophysiology and neuroimaging. The use of neuroimaging is growing continuously as an adjunct for estimating neurologic prognosis and we discuss which imaging modality is better depending on the situation. Computed tomography (CT) and magnetic resonance imaging (MRI) are the most commonly used imaging modalities in emergency neurological setting.^1^ For most acute neurocritical care conditions, CT is more commonly performed than MRI as it is widely available, faster and cheaper and interventions can be initiated promptly.^2^

Though, there are some practical limitations that could impact the widespread use of MRI due to difficulty in transport of hemodynamically unstable patients to the MRI suite and relative contraindications from temporary pacing wires and pacemakers/defibrillators, most neurocritical care disorders rely on MR imaging for early and accurate detection, treatment as well as follow-up due to its enhanced sensitivity, superior soft tissue and contrast resolution.^3^

We aim to update a review of major imaging conditions that help in neurocritical care management. These include stroke, infections, traumatic brain injury, metabolic encephalopathies, intracerebral hemorrhage and tumors.

## SEQUENCES

Various MRI sequences are available for detection and characterization of different emergency conditions that present with similar neurological symptoms. Appropriate set of sequences needs to be chosen to suit individual requirement based on clinical signs and symptoms.^4^

- *T1W:* Shows anatomical details and in combination with T2W images, it is used to characterize lesions and assess degree of contrast enhancement.
- *T2W:* Pathologic processes such as tumors, edema, ischemia and demyelinating conditions are best appreciated as they produce bright signal.
- *FLAIR (Fluid Attenuation Inversion Recovery):* Suppresses CSF signal effects and helps in detection of subtle white matter changes or edema. FLAIR is more sensitive for early detection of ischemic changes than T2W sequence. It is also the most sensitive MR sequence for detection of subarachnoid hemorrhage.
- *Diffusion weighted imaging (DWI):* It is based on the measurement of random Brownian motion of water molecules within brain tissues. DWI plays a critical role in detection of ischemic stroke as well as non-ischemic conditions such as infections, trauma, tumors and metabolic encephalopathy. DWI should be performed as the first sequence for determining irreversibly infarcted tissue core.
- *Gradient Recalled Echo (GRE) or Susceptibility Weighted Imaging (SWI):* Paramagnetic effects of blood products produce signal loss, called as ‘blooming’. These sequences are helpful in identifying all stages of bleed and microhemorrhages. They are commonly performed in patients with acute head trauma, hemorrhagic tumors and vascular malformations.
- *MR Angiography and Venography (MRA and MRV):* It can be done with or without contrast (time-of-flight angiography). It images both intracranial and extracranial vasculature to detect the site of arterial disease or venous thrombosis. Dynamic depiction of vessels helps in imaging work-up of slow and fast flow vascular malformations.
- *Magnetic Resonance Spectroscopy (MRS):* It is not usually done in emergency setting but provides information on metabolites in abnormal tissue and is useful for evaluation of tumors, demyelinating diseases and infections.
- *Perfusion:* Quantifies amount of blood flowing into tissue and generates values such as cerebral blood volume, cerebral blood flow and mean transit time. It is not routinely performed in emergency setting, but can help in defining the salvageable ischemic penumbra in ischemic stroke, assessing grade of certain tumors, or distinguishing radiation induced necrosis from tumor progression.

## ISCHEMIC STROKE

The aim of imaging in the acute phase of a suspected stroke is to exclude the presence of intracranial hemorrhage for which CT plays the pivotal role. MR helps in assessment of presence, location and extent of ischemic changes. This can be easily and accurately achieved with a fast (<15 minutes) MRI stroke protocol. The MR sequences typically used in the evaluation of acute stroke include DWI which is most sensitive as compared to the standard MRI sequences (T1W, T2W, FLAIR, GRE and MR angiography) (Flow chart 1).^5^

MRI has superior sensitivity and specificity in the diagnosis of acute ischemic infarction. It can also accurately detect secondary intracranial hemorrhage at various stages. Signal changes in different sequences also serve to age the infarction which has implications in the management and course of the disease.^1^

MRI also helps in the detection of pathologies that may resemble stroke clinically called “stroke mimics” such as vascular malformations, hemorrhagic tumors, infections, inflammatory diseases, metabolic or hypertensive encephalopathy. The distinction between ischemic stroke following arterial occlusion and ischemia due to venous sinus occlusion with secondary hemorrhage can also be made.

**Flowchart 1:**
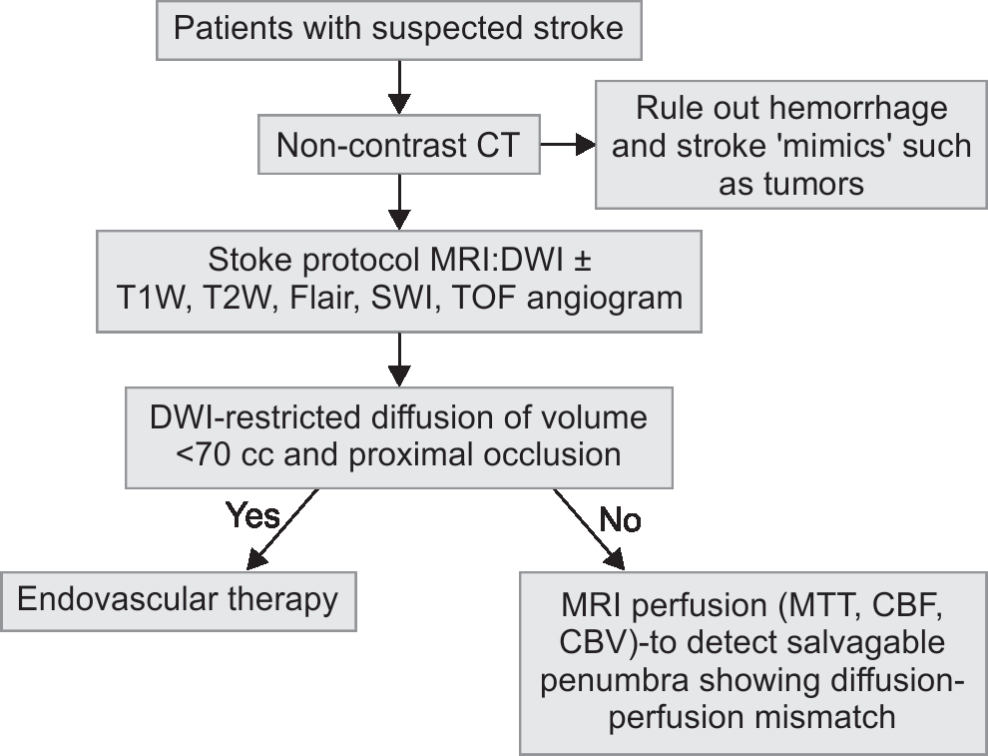
Imaging algorithm for acute ischemic stroke

**Fig. 1:**
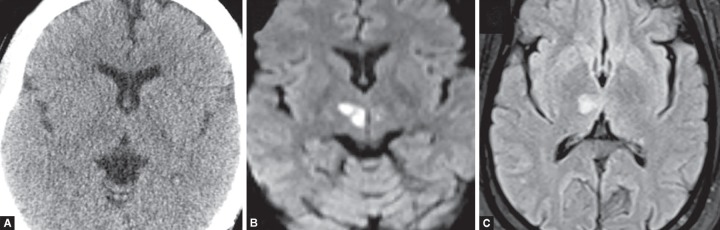
Acute right thalamic infarction not appreciated on CT. (A) but very well on MRI. It shows (B) diffusion restriction on DWI and (C) hyperintensity on FLAIR in the affected right thalamus

**Figs 2A to D:**
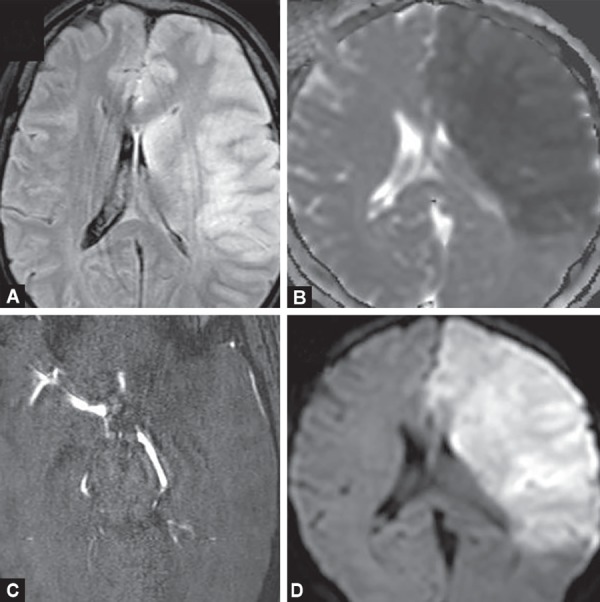
Large acute left MCA territory infarct appearing hyperintense on FLAIR (A) and showing diffusion restriction; (B) Loss of flow related enhancement in the left MCA in the TOF angiogram; (C) suggests occluding thrombosis (D) with low ADC values on ADC map

**Figs 3A and B:**
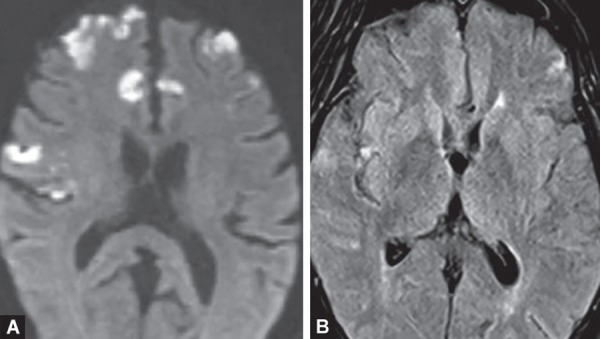
DWI sequence is more sensitive in detecting acute early age embolic infarcts which are not very well appreciated on FLAIR sequence

Changes of acute ischemic injury are detectable sooner with diffusion-weighted imaging of higher magnetic strength (Fig. 1). DWI is highly sensitive (88–100%), specific (95–100%), and accurate (95%) for detecting and delineating ischemic brain which has infarcted. Restricted diffusion seen as bright signal areas with corresponding signal drop on ADC within minutes following the onset of ischemia (Figs 2 and 3).

T2W/FLAIR hyperintensity with loss of gray matter-white matter differentiation, mass effect and sulcal effacement is shown by the infarcted tissue due to cytotoxic edema.

GRE or SWI sequences can detect small intracranial hemorrhage/hemorrhagic conversion of infarcted areas as well thrombosed vessels better than CT.

Intravascular thrombus in the affected territories is seen as loss of arterial flow voids on conventional T1W/T2W sequences which is better seen on time-of-flight MRA and contrast-enhanced MRA. MR angiography also helps in locating vascular thrombus, aneurysm and vascular malformations.

Perfusion-weighted imaging may be used to identify areas of reversible ischemia. Region that shows “matching” diffusion and perfusion abnormalities represents irreversibly infarcted core tissue, while a region that shows only perfusion abnormalities and has normal diffusion likely represent viable ischemic tissue, or a “penumbra” which can be salvaged by reperfusion therapy. Patients with small infarct core volumes (<70 mL) derive the greatest benefit after successful reperfusion in contrast to those with large initial infarct core volumes (>100 mL) which are at increased risk of hemorrhage after reperfusion and are unlikely to benefit from intra-arterial therapy. Infarcts between 70 mL and 100 mL at presentation are uncertain to benefit from intra-arterial therapy (IAT). Thus, evaluation of MR images at very early stage of stroke may help predict the clinical outcome.

### Traumatic Brain Injury

CT is the modality of choice during the first 24 hours after the injury as it easily detects bone fractures and bleed such as acute epidural hematoma, subdural hematoma, subarachnoid hemorrhage and hemorrhagic cerebral contusions.^6^

**Fig. 4:**
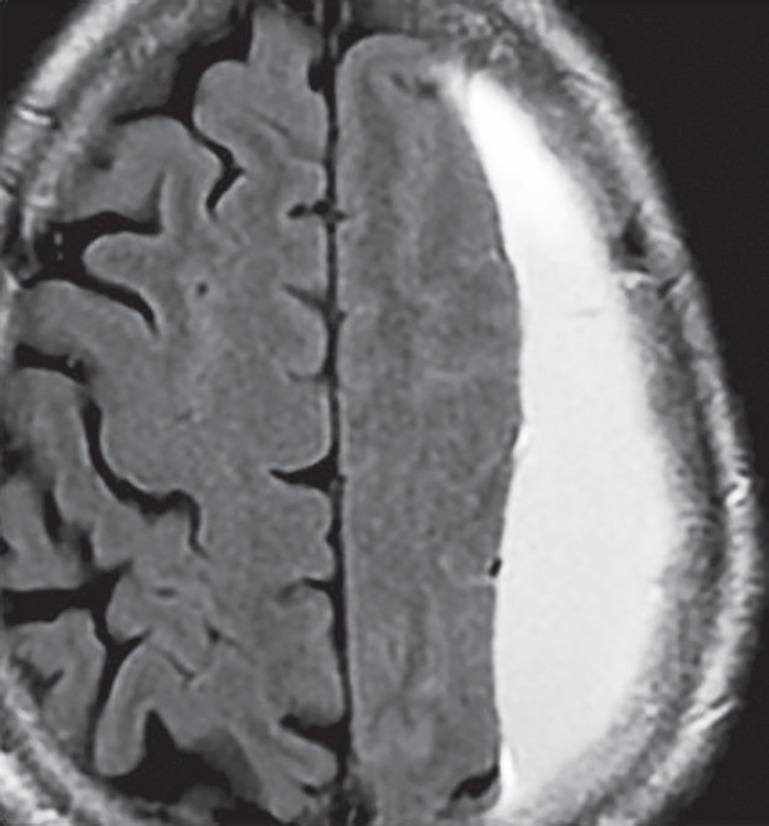
Subdural hematoma along left cerebral convexity appearing hyperintense on T1W image suggestive of subacute phase of hematoma

However, MRI is more sensitive and accurate in diagnosing neuronal damage in acute traumatic brain injury such as diffuse axonal injury, cortical contusions and brainstem injury. It also has better ability to detect subacute to chronic hematomas as the composition of the blood changes over time (Fig. 4).^7^

**Fig. 5:**
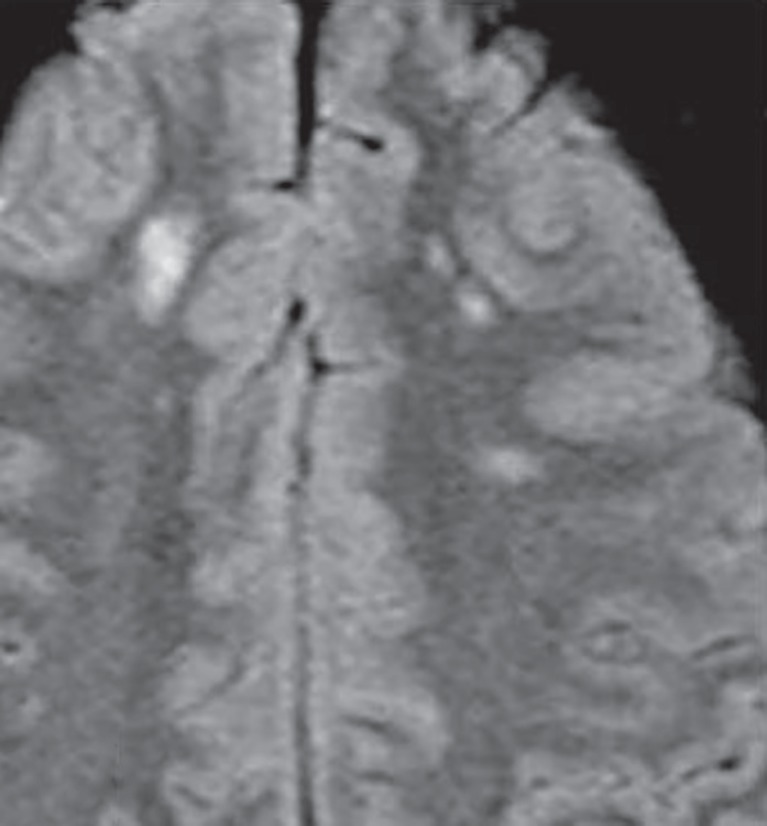
Diffuse axonal injury (DAI). Punctate FLAIR hyperintense foci in subcortical and deep white matter which are difficult to pick up on CT scan images in post trauma setting

Diffuse axonal injury is caused by shearing of axons due to rotational forces. Initial CT imaging may be normal but follow-up MRI shows T2W/FLAIR hyperintense areas in subcortical white matter at the gray-white junction, mainly in frontotemporal lobes followed by corpus callosum and dorsolateral brain stem (Fig. 5). They may contain microhemorrhage showing susceptibility artifact on GRE or SWI imaging. Diffusion-weighted sequence play a crucial role as it can detect acute areas of non-hemorrhagic DAI showing diffusion restriction, that may not be detectable on T2W and FLAIR images.^8^

**Figs 6A and B:**
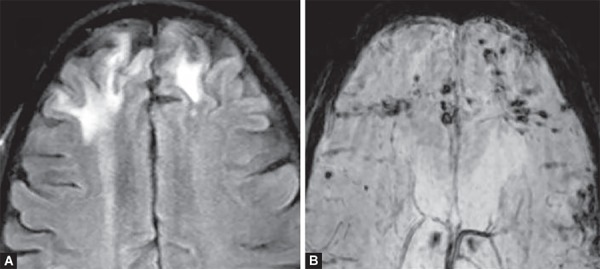
FLAIR hyperintense (A) areas of post traumatic contusions in bilateral frontal lobes, with multiple small hemorrhagic contusions picked up on SWI image (B) due to susceptibility blooming artifact secondary to hemorrhagic areas

FLAIR sequences may find small acute or subacute SAH and epidural bleeds, missed by CT. Susceptibility weighted imaging is useful for detecting microhemorrhages in contusions (Fig. 6).

MRI can be used to assess secondary injuries such as edema causing mass effect, midline shift, compression on vascular structures resulting in infarct, or brain herniation.^6^

MRA can provide information about the arterial walls which can be disrupted due to trauma, leading to dissections, aneurysms or fistulae.

Though CT is the imaging modality for skull base fractures, MRI can be used to precisely identify site of CSF leaks, with the help of DRIVE/CISS sequences.

### Cerebral Herniation

It commonly presents secondary to trauma or intracranial neoplasms and may lead to compression of vital structures, vasculature, and cranial nerves. MRI due to its better soft tissue definition and multiplanar imaging is superior in detecting transtentorial and subfalcine herniations. Beam hardening artifacts from the skull base bones can interfere with CT interpretation of tonsilar herniations, which is not the case in MRI.

### Spontaneous Intracerebral Hemorrhages (ICH)

Common etiologies presenting in acute setting are hypertension, cerebral amyloid angiopathy, vascular malformations, aneurysms, tumors, cerebral venous thrombosis, hemorrhagic transformation of an ischemic infarct, brain tumors and vasculitis.

**Figs 7A and B:**
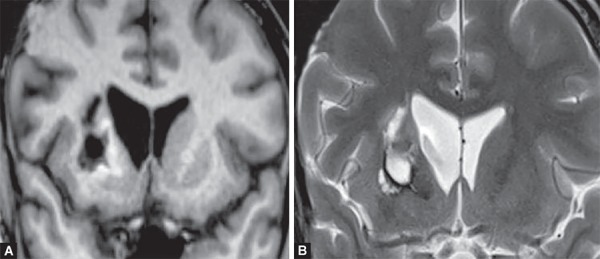
Chronic hypertensive intraparenchymal bleed appearing as hypointense on T1 (A) bright on T2W (B) due encephalomalacic changes and hemosiderin stained rim in the right basal ganglia

While CT is superior to detect arterial vascular lesions and active bleeding, MR imaging is far more superior at detecting non-arterial causes and detection of age of hemorrhage (Table 1). Chronic blood and microhemorrhages (i.e., hemorrhages <10 mm in size) are seen better using GRE and SWI sequences (Fig. 7).^9^

Hypertension, the most common cause of spontaneous brain hemorrhage, has a predilection for the deep brain structures because it is believed that small penetrating arteries that arise directly off of medium-caliber vessels have high perfusion pressures which will be more exaggerated in the setting of hypertension. The caudate and putamen are its most common locations, followed by the thalamus, cerebellum, and pons.

Underlying brain tumor is an important cause of secondary ICH, which can occur in up to 10% of primary and metastatic tumors. Primary brain tumors such as glioblastoma and oligodendroglioma and metastasis from renal, thyroid, and choriocarcinomas are associated with the greatest frequency of bleed. Large ICH can obscure underlying tumor, which can be detected separately from the bleed on contrast enhanced MRI.^10^

**Table 1:** Temporal evolution of intracranial bleed on MRI. Grey colour indicates isointense signal to the brain parenchyma, white indicates hyperintense and black represents hypointense signal.

*Stage*	*Age*	*Hemoglobin*	*T1W SI*	*T2W SI*
Hyperacute	<24 hrs	Oxyhemoglobin	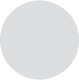	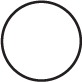
Acute	1–3 days	Deoxyhemoglobin	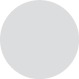	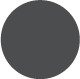
Early subacute	>3 days	Intracellular methemoglobin	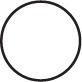	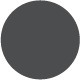
Late subacute	>7 days	Extracellular methemoglobin	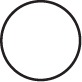	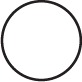
Chronic	>14 days	Hemosiderin	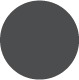	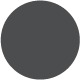

**Fig. 8:**
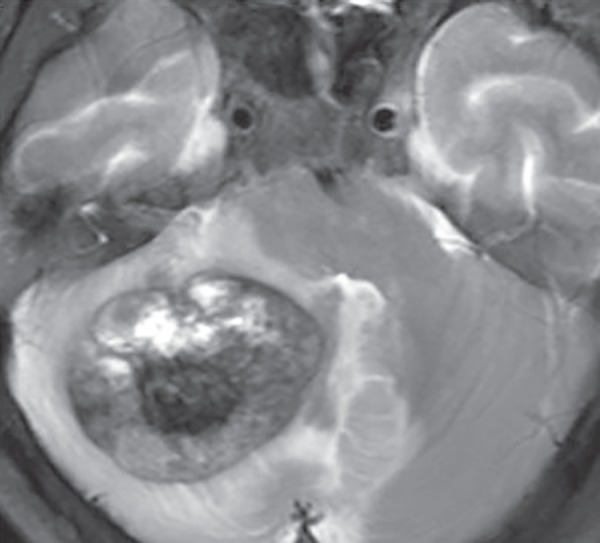
Giant cavernoma containing mixture of signal intensities on T2W image due to blood in different stages. Surrounding parenchyma edema is seen due to recent bleed

MRI is much more sensitive for detecting small cavernous malformations, which frequently show a “popcorn” appearance on T2W images, due to blood products in various stages of degradation, also readily visible on GRE and SW sequences (Fig. 8).

### Arteriovenous Malformation (AVM)

MRI is more sensitive to detect the presence and delineate the extent of AVM and aneurysms even in the background of hemorrhage. Hemorrhage and its mass effect can obscure delineation of an AVM on CT.^11^ It is also useful in early detection of secondary effects caused by AVMs such as brain infarcts.

**Figs 9A to D:**
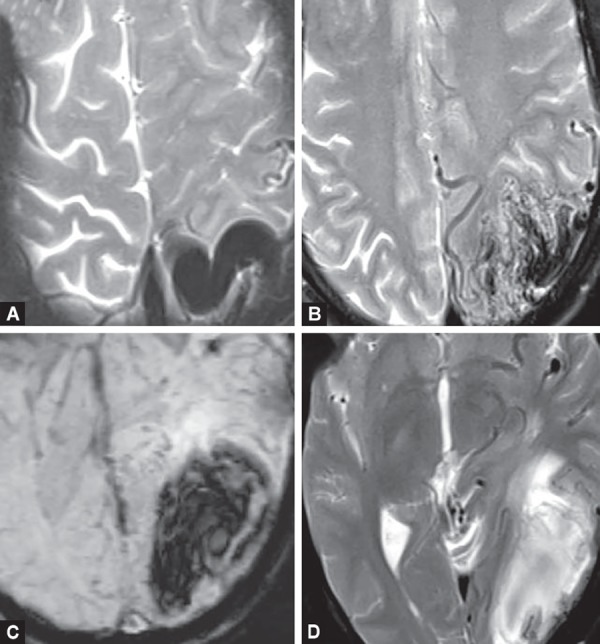
AVM. Multiple dilated and tortuous feeding arteries and draining vein seen as “flow voids“around the nidus in a wedge-shaped configuration. (A and B). T2W hyperintense (D) area of hemorrhage is seen around it and showing ‘blooming’ on SWI images(C) appears to be in subacute to chronic stage

Dynamic contrast-enhanced MR angiography provides information about hemodynamics of vascular malformations, differentiating between high and low flow malformations and best depict the enlarged feeding arteries and draining veins appearing like “bag of worms”.^12^ Fast and turbulent flow in the abnormal vessels and nidus produce tangle of signal flow voids seen on T2W images. Blood-sensitive sequences such as GRE can detect the presence of hemosiderin staining, indicating old hemorrhage (Fig. 9).^13^

**Figs 10A and B:**
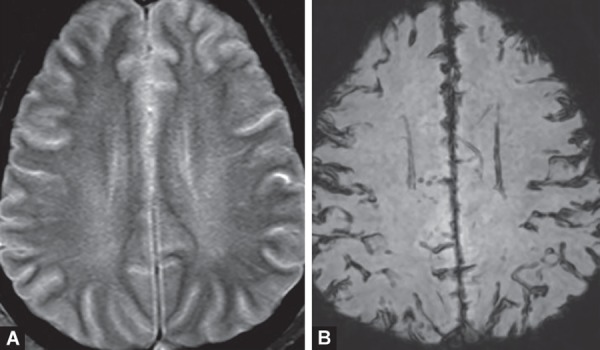
Subarachnoid bleed in all sulcal spaces difficult to appreciate on the routine T2 W image (A) and shows appears hypointense on SWI(B) due to susceptibility artifact

### Subarachnoid Hemorrhage (SAH)

Non-contrast CT is the initial diagnostic test for suspected SAH and the sensitivity reaches upto 100% when performed within 6 hours but decreases by 10–15% with time as blood is progressively diluted by normal cerebrospinal fluid (CSF) flow. These patients should be further evaluated with CT angiography and negative CT angiography will be evaluated with MR imaging, especially patients with peripheral convexity SAH (Fig. 10).

FLAIR sequence is sensitive in the acute phase and shows sulcal hyperintensity. T2* sequences have sensitivity of 94% within 4 days but reaches 100% sensitivity later with a high specificity.

### Cerebral Venous Thrombosis

It is a neurologic condition that presents with features of increased intracranial pressure (headache and confusion), infarction or hemorrhage due to venous outflow obstruction.

Conventional MR sequences, unenhanced TOF venography, GRE and contrast-enhanced MR venography have greater sensitivity than CT scan to detect venous sinus and cerebral venous thrombosis and associated brain parenchymal changes.^14^

Thrombosed veins and sinuses appear hyperintense on conventional T1W/ T2W sequences and hypointense on GRE because of methemoglobin. TOF MRV showing loss of signal is the most commonly used method for evaluation of thrombosis because of its excellent sensitivity to slow flow. Contrast enhanced MR venogram may be required for confirmation as it improves visualization of filling defects, recanalization changes and dural collaterals.

**Figs 11A to C:**
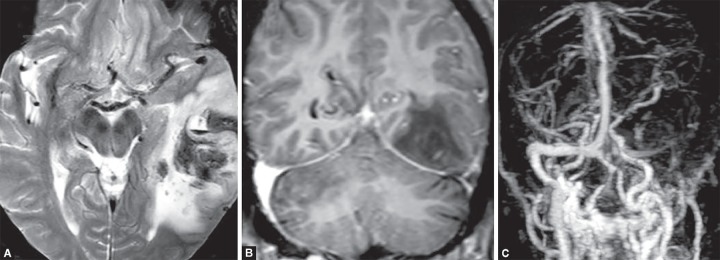
Venous infarct with secondary hemorrhagic transformation. (A) Left temporal lobe shows heterogeneous parenchymal bleed with surrounding edema on T2W image due to thrombosed left transverse sinus. (B) The sinus shows no contrast filling and (C) shows loss of flow related enhancement on 3D TOF image

Parenchymal changes such as edema, sulcal effacement, and ventricular narrowing due to vasogenic and cytotoxic edema are also seen. Parenchymal hemorrhage appears as flame-shaped areas of lobar hemorrhage in cortical and subcortical locations (Fig. 11).

### CNS Infections

Majority of the central nervous system (CNS) infections presenting in the emergency setting are secondary to bacteria and virus and less commonly due to fungus or parasitic etiology.

Bacterial infections of the CNS are neurologic emergencies. These commonly manifest as meningitis, vasculitis, infarctions, brain abscess, empyemas, and suppurative dual sinus thrombophlebitis. Though the “gold standard” for the diagnosis of bacterial meningitis is CSF analysis, MRI can depict both primary manifestations of meningitis as well as secondary complications.

**Acute meningitis** shows purulent exudates in basal cisterns and superficial sulci, which appear isointense with brain on T1WI and T2W images and do not suppress on FLAIR giving the appearance of “dirty” CSF. Post contrast sequence is mandatory as it clinches the diagnosis in the appropriate clinical scenario. Leptomeningeal enhancement is typical for meningitis.

**Figs 12A to C:**
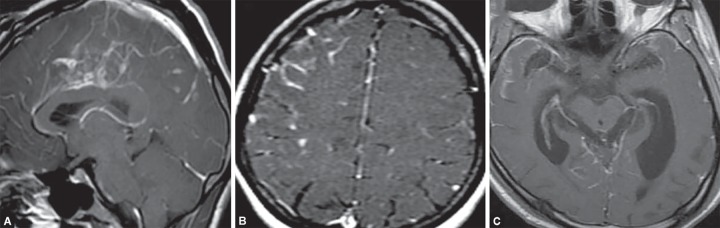
(A) Bacterial meningitis; (B) Diffuse leptomeningeal and pachymeningeal enhancement along cerebral convexities and basal cisterns; (C) Hydrocephalous secondary to meningitis

MR imaging is the modality of choice for detection of complications of meningitis such as hydrocephalus, subdural effusion, subdural empyema, ventriculitis, cerebritis, cerebral abscess, venous thrombosis and ischemia (Fig. 12).

**Figs 13A to C:**
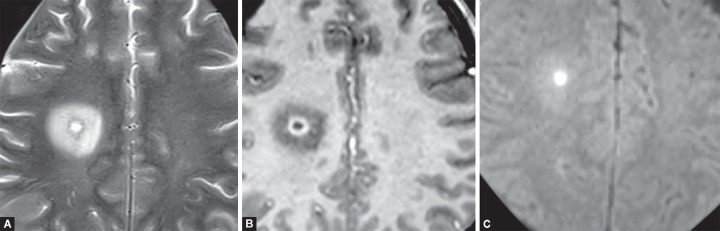
Cerebral abscess. (A) Ring enhancing lesion; (B) in right centrum semiovale of right frontal lobe with T2W hyperintense core, perilesional edema; (C) and central diffusion restriction on DWI image

**Cerebral abscess** presents as centrally T2W/FLAIR hyperintense lesion with peripherally enhancing capsule and surrounding parenchymal edema. DWI is the most useful diagnostic marker as abscesses show central diffusion restriction due to thick purulent material, which differentiates them from necrotic tumors that do not show restricted diffusion (Fig. 13).^15^

**Figs 14A to C:**
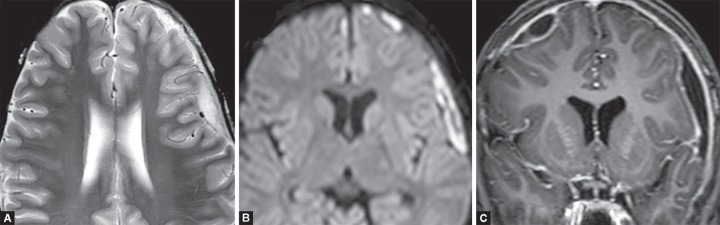
Subdural empyema. (A) T2W hyperintense collection in subdural space showing (B) restricted diffusion and (C) thick rim enhancement

Empyemas present as extra-axial purulent collection in subdural or epidural space, appearing iso-hyperintense compared with CSF on T1W, T2W and FLAIR sequences and showing thick enhancing encapsulating membranes (Fig. 14).^15^ Diffusion sequence plays an important role in confirming the diagnosis, as the pus shows strong diffusion restriction.

**Acute viral encephalitis,** commonly due to herpes simplex virus can promote acute fulminant CNS disease and present as neurocritical emergency.

It commonly affects bilateral limbic system (anterior and medial temporal lobes, insular cortex, inferior frontal lobes, and cingulate gyri) in asymmetric manner.

MR is again the modality of choice and shows gyral swelling presenting as T2W/FLAIR cortical/subcortical hyperintensity with indistinct gray-white junction and diffusion restriction. FLAIR is the most sensitive sequence and is the first sequence to show positive findings.

**Figs 15A to D:**
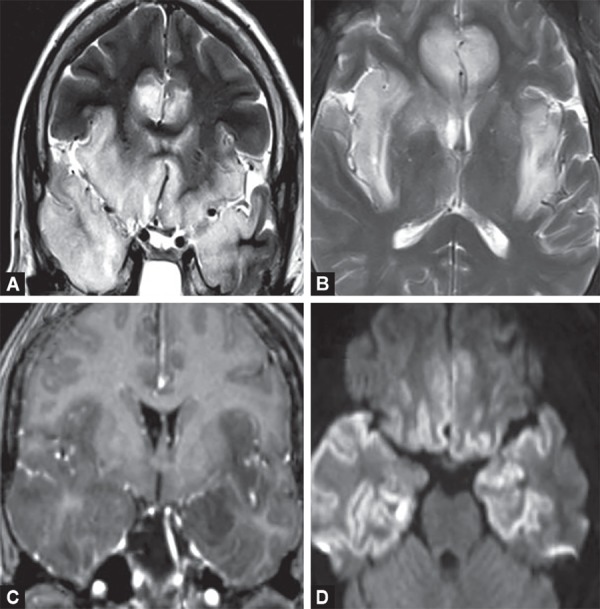
HSV Encephalitis. Asymmetric hyperintensity and swelling in bilateral temporal lobes, insular region and basifrontal lobes on T2W (A and B) images. (C) No contrast enhancement on T1 post contrast images. (D) Diffusion restriction in the affected region.

T2* (GRE, SWI) imaging may demonstrate hemorrhages. No enhancement may be seen in early phase (Fig. 15).^15^

**Figs 16A to C:**
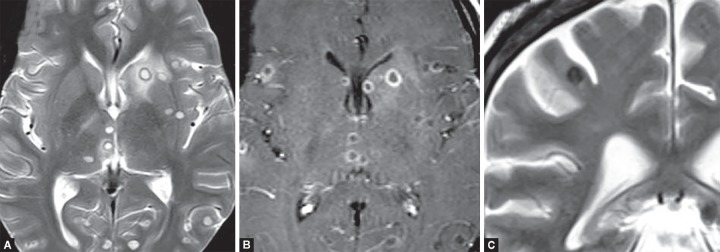
Neurocysticercosis. Colloid vesicular stage (A and B) showing multiple ring enhancing lesions, appearing centrally bright on T2W and showing peripheral edema (C) Dark calcified nodule without edema in nodular calcified stage

**Neurocysticercosis** presents with seizures and neurological deficits. Imaging findings depend on the stage of infection, number of lesions and host inflammatory response at presentation. Vesicular stage shows features of a thin-walled cyst that is similar to CSF. Scolex is best appreciated on MRI as a nodular “dot in a hole” appearance. In colloidal vesicular stage, the cyst shows enhancing wall and marked surrounding edema. Cerebral edema decreases in granular nodular stage. Nodular calcified (inactive) stage shows a small calcified nodule, better seen on CT than MRI (Fig. 16).^15^

**Figs 17A and B:**
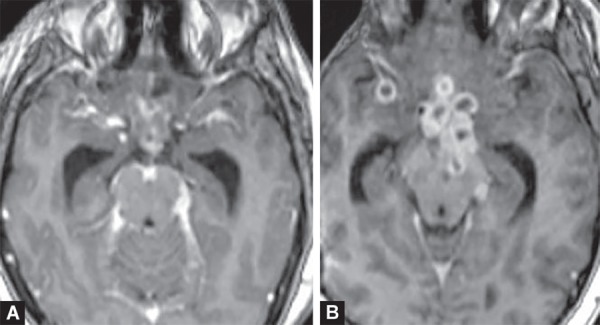
Tuberculous meningitis. (A) Thick enhancing exudates along basilar cisterns and sylvian fissures. (B) Clustered ring enhancing tubercular abscesses

**Tuberculosis** is the most endemic neurological infection in our country. It presents with several manifestations such as acute meningitis, tuberculoma, abscess, vasculitis, infarction and hydrocephalus. Exudates in the sulcal spaces and basal cisterns appear hyperintense on FLAIR along with nodular and thick enhancement of the basal cisterns, sylvian fissure and meninges (Fig. 17).^16^

TB abscesses are usually hyperintense on T2W/FLAIR with a T2W hypointense enhancing ring resembling pyogenic abscesses. MR spectroscopy shows elevated lipid peaks due to high lipid content of the mycolic acid within the bacteria.

### Brain Tumors

Patients with brain tumors can present with emergency with features either related to the growth of a known primary tumor itself or secondary to its complications such as worsening edema, herniation, infections (due to immunocompromised state), secondary hemorrhage or infarction due to tumor emboli.^17^ Primary and metastatic brain tumors exert mass effect and vasogenic edema causing increase intracranial pressure. Patients may present with post treatment toxicity due to chemotherapeutic drugs or post radiation effects. MRI is the modality of choice to differentiate pseudoprogression due to radiation necrosis from true disease progression.

Communicating hydrocephalus requires screening of the entire neuroaxis to look for leptomeningeal carcinomatosis. Obstructive hydrocephalus can occur due to lesions obstructing the ventricular system, commonly due to pineal or posterior fossa mass.

**Figs 18A to D:**
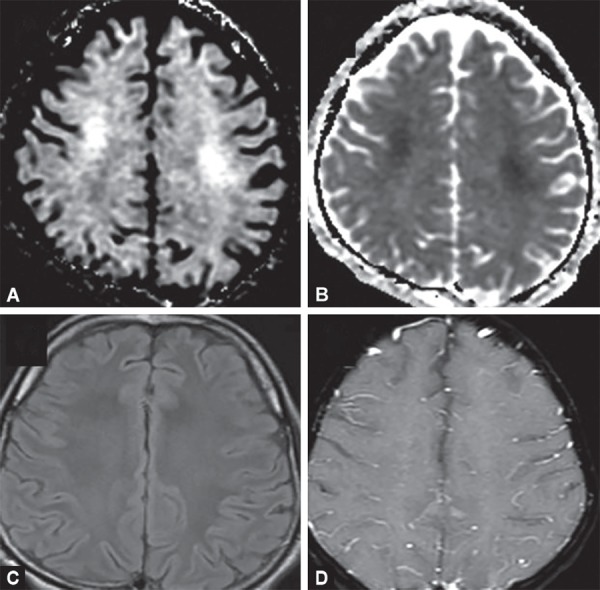
Methotrexate toxicity. Symmetric diffusion restriction in high frontal white mater (A and B) is the earliest change on imaging, where as FLAIR (C)and contrast images (D) are normal

## METABOLIC ENCEPHALOPATHY

### Methotrexate Toxicity

Methotrexate is a main component of chemotherapeutic regimens for acute lymphoblastic leukemia. It can cause acute neurologic symptoms usually within few weeks of starting therapy, either intravenously or intrathecally. Imaging findings include T2W/FLAIR hyperintensities in deep periventricular white matter showing diffusion restriction due to cytotoxic edema which are usually reversible. No contrast enhancement is seen (Fig. 18).

### Reversible Posterior Leukoencephalopathy Syndrome (PRES)

**Figs 19A to D:**
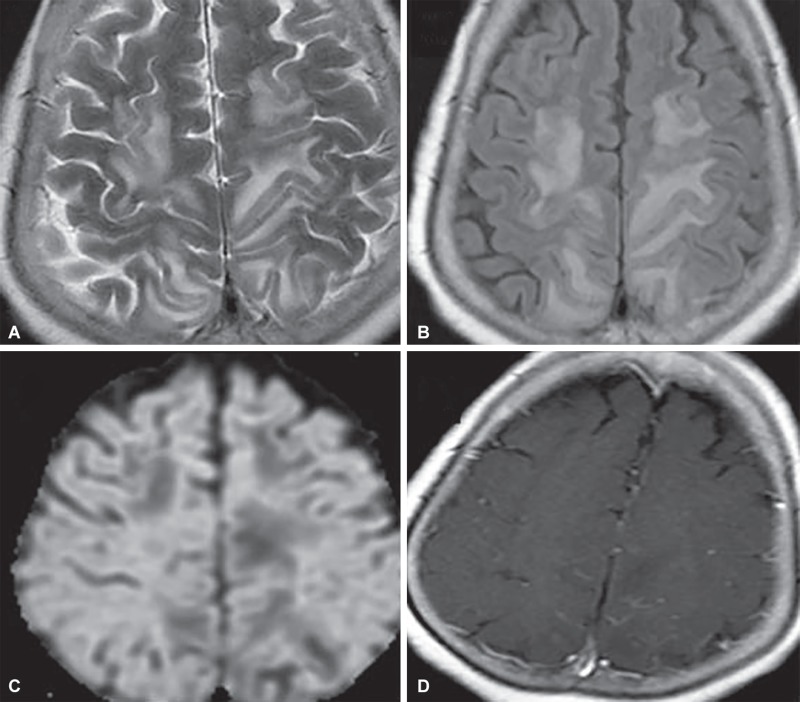
Posterior reversible encephalopathy syndrome. Symmetrical T2W (A) and FLAIR (B) hyperintensity in parieto-occipital subcortical white matter. No diffusion restriction (C) or contrast enhancement (D) helps to differentiate from infarctions

It is a neurotoxic state presenting to the emergency with seizures, visual changes and altered mental status in the background of high blood pressure. The parietal and occipital lobes are most commonly involved, followed by frontal lobes and cerebellum. Brain stem and basal ganglia may also be involved. Vasogenic edema is noted at affected locations and is symmetric and reversible on follow-up imaging. The affected areas appear T2W/FLAIR hyperintense, do not enhance with contrast and do not show diffusion restriction. Atypical cases may show post contrast enhancement of adjacent cortical vessels and restricted diffusion (Fig. 19).

### Demyelinating Disease

#### Central Pontine Myelinolysis

**Figs 20A to C:**
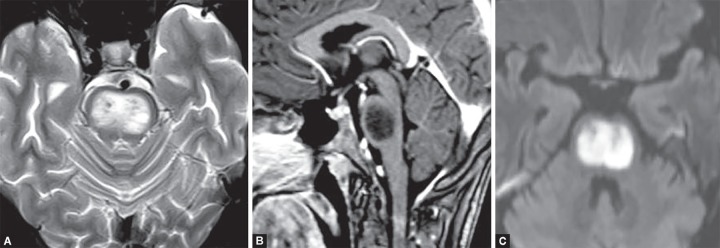
Central pontine demyelination. (A) T2W hyperintense signal in central part of pons with intense diffusion restriction; (B) The peripheral fibres of pons are spared; (C) No post contrast enhancement

It is a demyelinating process involving the central part of pons, and less commonly the basal ganglia and cerebral white matter. The patients present with seizure and encephalopathy due to electrolyte imbalance usually in the setting of rapid correction of hyponatremia. The affected areas appear hyperintense on T2W/FLAIR, show intense diffusion restriction and no contrast enhancement (Fig. 20).

#### Tumefactive Demyelination

They manifest as tumor-like lesions appearing as T2/FLAIR hyperintense white matter lesions, size more than 2 cm, showing edema and incomplete ring enhancement. Rim of restricted diffusion may be seen. MRI helps in early detection which can lead to early initiation of steroids and also helps to differentiate from gliomas and abscesses.

### Acute Spinal Injury

**Figs 21A and B:**
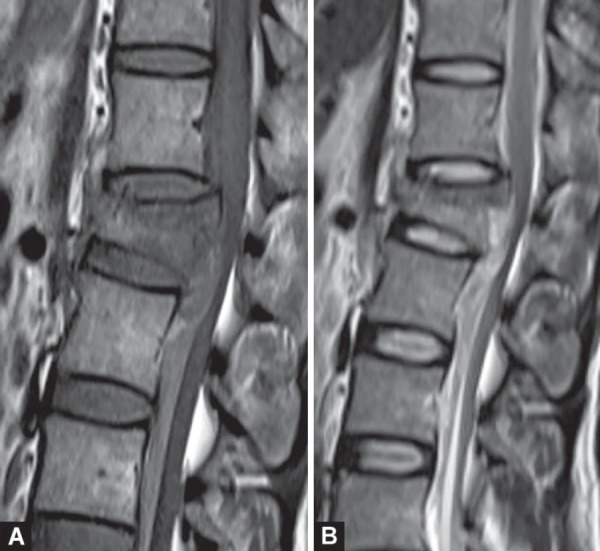
Spinal cord injury. Burst fracture and retropulsion of thoracic vertebral body compressing the spinal cord and producing spinal cord edema. T1W (A) and T2W (B) hyperintense epidural hematoma noted anterior to the cord

MRI is the modality of choice for evaluation of patients with neurological signs or symptoms suggestive of cord injury. Spinal cord injury may be due to direct compression from bony fracture fragments, epidural hematoma, or herniated disc material. It may present with edema appearing T2W/STIR hyperintense along the length of injured cord with or without hemorrhage whose signal depends on the stage of blood. MRI also helps in evaluating the severity and extent of cord compression and other associated soft tissue injuries (Fig. 21).^18^

### Spinal Epidural Hematoma

It commonly presents as acute onset of neurologic impairment in postoperative or post traumatic setting. It appears as a collection in epidural space, appearing iso-hyperintense on T1W imaging and hyperintense on T2W imaging depending on age of blood products. No post contrast enhancement is seen. Mass effect causing direct compression of the spinal cord or nerve roots may also be noted (Fig. 21).^18^

**Fig. 22:**
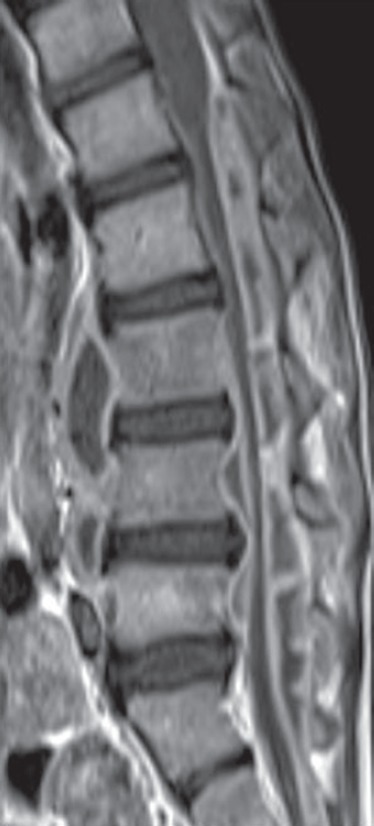
Spinal epidural empyema. Thick-walled collection in both anterior and posterior epidural space of lower spinal canal, causing narrowing of spinal cord. Enhancing septae are seen within. Prevertebral abscess also noted

**Figs 23A and B:**
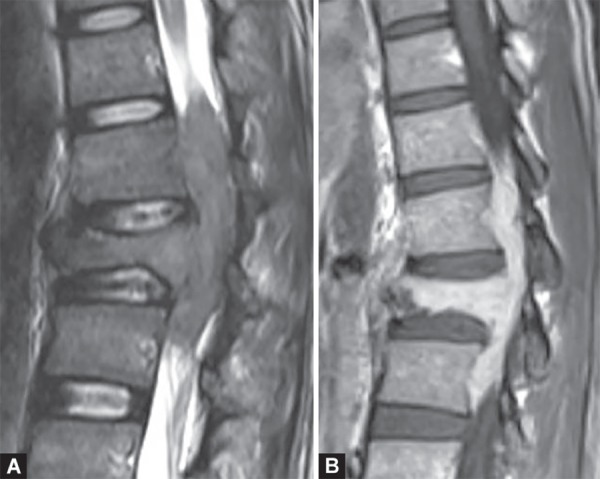
Vertebral metastasis seen as T2W isointense (A) and enhancing; (B) soft tissue mass extending into epidural space and causing cord compression. Collapse of vertebral body also noted

**Figs 24A and B:**
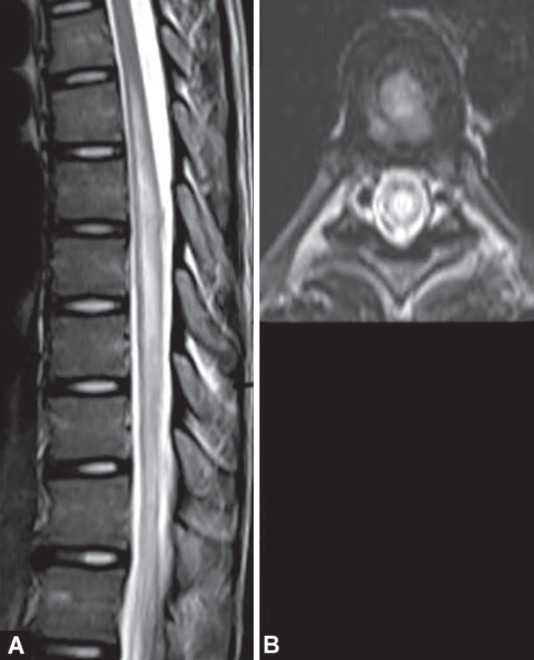
Transverse myelitis. Long segment cord expansion with central cord T2W hyperintense signal involving the thoracic cord

### Spinal Epidural Abscess

Pus in the extradural space may cause direct compression of the spinal cord or lead to infarcts due to occlusion of spinal cord vascular supply. It appears hyperintense on T2W imaging, shows restricted diffusion and rim enhancement, similar to cerebral abscess. It may be associated with vertebral osteomyelitis (Fig. 22).^18^

### Spinal Metastasis

MRI is the gold standard for assessment of effects on spinal cord due to metastatic spine disease. Metastasis can present as soft tissue lesions most commonly extending from vertebrae and are located in extradural space of spinal canal, leading to cord compression and producing cord edema (Fig. 23). They may also present as nodular or diffuse leptomeningeal disease along the spinal cord surface.^17^

### Inflammatory Myelopathy

**Acute transverse myelitis** is an inflammatory condition presenting with sudden onset bilateral motor, sensory and autonomic abnormalities. It manifests as central T2W hyperintense signal occupying more than two-thirds of the cross-sectional area of the spinal cordand extending over three to four segments, usually affecting the thoracic spine. No enhancement or diffusion restriction may be seen. Spinal cord expansion is seen along affected length of the cord (Fig. 24).

MR imaging in the emergency setting can give a quick and precise diagnosis of various neurological conditions and should be performed in suitable conditions. MRI sequences play individual roles to provide information contributing to the clinical diagnosis and thus helping in prompt patient management.
